# Detailed characterization of plant-based burgers

**DOI:** 10.1038/s41598-021-81684-9

**Published:** 2021-01-21

**Authors:** Massimo De Marchi, Angela Costa, Marta Pozza, Arianna Goi, Carmen L. Manuelian

**Affiliations:** Department of Agronomy, Food, Natural Resources, Animals and Environment, University of Padova, Viale dell′Università 16, 35020 Legnaro, PD Italy

**Keywords:** Biochemistry, Plant sciences, Zoology

## Abstract

Flexitarians have reduced their meat consumption showing a rising interest in plant-based meat alternatives with ‘meaty’ characteristics, and we are witnessing an unprecedented growth of meat substitutes in the Western market. However, to our knowledge, no information regarding the ‘simulated beef burgers’ nutritional profile compared to similar meat products has been published yet. Here we show that, whilst both plant-based and meat-based burgers have similar protein profile and saturated fat content, the former are richer in minerals and polyunsaturated fatty acids. We found that the most abundant minerals in both categories were Na, K, P, S, Ca, and Mg; being Na and S content similar between groups. Only six amino acids differed between categories, being hydroxyproline exclusively in meat-based burgers. Plant-based burgers revealed fourfold greater content of n-6 than meat-based burgers, and greater short-chain fatty acids proportion. Our results demonstrate how ‘simulated beef’ products may be authenticated based on some specific nutrients and are a good source of minerals. We believe that there is a need to provide complete and unbiased nutritional information on these ‘new’ vegan products so that consumers can adjust their diet to nutritional needs.

## Introduction

The neologism “flexitarian” has been recently adopted in scientific and public sectors to refer to those meat consumers who recognize meat as an important source of some nutrients (protein, fat, and micronutrients) but who also take into account ethical criteria (e.g. sustainability in intensive production, and animal welfare)^1^. Thus, flexitarians have significantly reduced their daily meat consumption^1^. Meat-like products in Western markets, such as tofu and textured soy protein products, started in the early 1960s^2,3^, but consumers’ interest in plant-based meat alternatives has started to rise only recently^2,3^. More important, it seems that the target population for these products has moved to a more mainstream audience^3,4^, with products resembling burger patties, mince, sausages, and strips, specifically designed to exhibit ‘meaty’ characteristics appealing to meat-eaters^3,5^. Currently, there is not a universal regulation regarding the naming of meat substitutes, in contrast with dairy products whose terms and names are already protected by a European Union law^6^. This could give meat consumers the wrong impression that the nutritional profile of those products mirrors animal-based meat^3^; especially, since meat-related words and imagery is used to promote them^3^. Flexitarian consumers expect that the intake of plant-based products will help them reduce the intake of saturated fat and cholesterol associated with meat consumption, and will provide phytochemicals and fibers, characteristics which are conventionally considered desirable in the diet^7^. However, relating disease outcomes to meat intake is misleading, because a high consumption of meat could indicate a low intake of fish, fruit, and vegetables, and can be linked to unhealthy lifestyle factors such as lack of physical activity, smoking, imbalanced diet, and overeating^8^. Moreover, a direct causal relationship between meat consumption per se and onset of cardiovascular disease has not been proved and most lipid researchers agree that dietary cholesterol is not among major risk factors for elevated blood cholesterol and cardiovascular issues^8^. Also, the role of saturated fats in the development of heart disease is still unclear^8^. Despite the unprecedented growth that meat substitutes have recently experienced in the European markets^9^, little has been published regarding their nutritional composition compared to similar meat products. Therefore, this study compared the nutritional composition of meat-based burgers (**MBB**) and plant-based burgers (**PBB**) currently available in the supermarkets of the European Union.

## Results and discussion

### Color, pH, gross composition, and cooking loss

Color is an economically relevant quality trait as it affects sensory perception and consumers’ acceptance of foods, and pH has a significant effect on pigments responsible for the color of fruits, vegetables, and meat^10^. The raw PBB analyzed presented a slightly higher and more variable pH than the raw MBB, likely due to the greater alkalinity and diversity of the ingredients used to manufacture PBB (Table 1). These results are in line with those reported in a recent study^11^ comparing chicken sausages with plant-based sausages manufactured with soy protein isolate. Consumers expect that raw burgers present a reddish color, because they associate the term ‘burger’ with a meat product, where the reddish color is related to the greater presence of hemoglobin –heme prosthetic group– due to erythrocytes and myoglobin^12^. However, legumes such as soy also contain some amount of symbiotic hemoglobin known as ‘leghemoglobin’^13^. That difference in hemoglobin content explains why MBB raw products showed greater green–red (a*) and blue-yellow (b*) color compounds than the PBB (Table 1). Nevertheless, both burgers categories had the same lightness (L*) (Table 1). Most of PBB currently available on the supermarket shelves –and included in the present study– have soy or pea protein and beets as ingredients, which help create the impression of ‘bleeding’^5^ to better imitate meat making these products more attractive for meat consumers.

**Table 1:** Analysis of pH and color (median and 95% CI_50%_^1^) of raw burgers.

Trait^2^	Meat-based burger		Plant-based burger		
	Median	95% CI_50%_	Median	95% CI_50%_	*p*
pH	5.48	5.28–5.70	5.81	5.58–7.29	0.038
L*	44.89	42.36–48.61	47.99	39.87–48.90	0.481
a*	19.82	16.95–20.94	16.83	15.60–17.45	0.032
b*	14.46	13.57–15.88	11.21	9.63–11.77	0.004

^1^95% CI_50%_: median 95% confidence interval.

^2^Color components: L*, lightness; a*, green–red; b*, blue-yellow.

Some differences in the gross composition were also observed (S-Table 1). The greater carbohydrates content (P = 0.003) in PBB (Median, 8.37% of the raw product) than in MBB (Median, 2.04% of the raw product) was likely related to the greater total dietary fiber in PBB (PBB Median, 4.27% of the raw product; MBB Median, 0.74% of the raw product; P = 0.003) and not to the difference in starch and fructose content (S-Table 1). A recent study conducted in major supermarkets^3^ of Sydney (Australia) revealed that plant-based products (burgers, sausages, and mince) include greater carbohydrates and dietary fiber content than their animal-based homonymous. Dietary fiber includes those plant components that escape digestion and absorption in small intestine such as non-starch polysaccharides and oligosaccharides (e.g. cellulose plants and seed extracts) and carbohydrate analogues (e.g. pea, vegetables, and legumes)^14^. In western diets it is recommended to increase the dietary fiber ingestion, thus PBB could help achieve this goal. In addition, the incorporation of dietary fiber in meat products reduces their cooking loss^14^; thus, the lower cooking loss observed in PBB (Median, 16.01%) compared to MBB (Median, 25.67%; P = 0.004) when using the water bath method can be explained by the greater content in dietary fiber in the former than in the latter (S-Table 2). Moreover, the greater moisture content (P = 0.037) of raw MBB (Median, 65.91%) compared to raw PBB (Median, 60.91%) could also explain the greater cooking loss observed in MBB (S-Table 1). A lower shear force was needed to cut PBB (6.34 N) than MBB (12.85 N; P = 0.004) when cooked using the water bath method (S-Table 2), probably due to the greater dietary fiber content of PBB. These findings were in agreement with results obtained in plant-based and chicken sausages^11^, where a significant lower cooking loss and a numerical lower shear force in the former than in the latter were reported. However, we did not observe such differences in shear force when the cooking plate method instead of the water bath one was used (S-Table 2).

### Minerals composition

**Figure 1:**
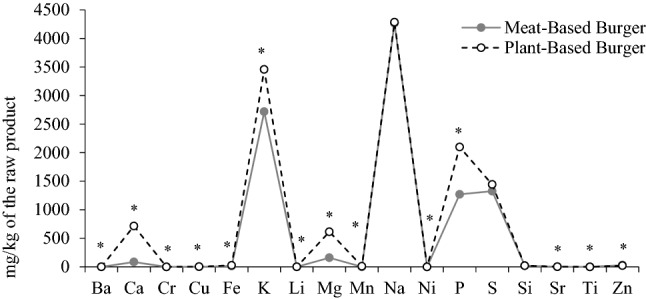
Mineral composition (median) of raw product (*p < 0.05).

Minerals in food gross composition are considered in the ashes content and they play a significant role in maintaining good health. Ashes content was greater (P = 0.003) in PBB (Median, 2.52% of the raw product) than in MBB (Median, 1.79% of the raw product) (S-Table 1), showing a clear different mineral profile (Fig. 1 and S-Table 3). In both groups, the most abundant minerals were Ca, K, Mg, Na, P, and S. Only Zn was less abundant in PBB than in MBB, and Na, S, and Si content was similar in both categories. Heavy metals such as Pb, As, and Hg were not detected; however, one of the PBB brands revealed Cd in all its four samples. The main food groups which contribute to Cd dietary intake are grains and vegetables due to soil contamination from agricultural practices (e.g., use of fertilizers)^15^. Nevertheless, Cd bioavailability of animal-based food may be greater than that of plant-based foods^16^. Also, B and Mo were only present in PBB; this was expected as they are plants trace elements. Lithium was detected in all four samples of the same PBB’s brand –the same that presented Cd–, and only in one sample of the MBB category. Despite its benefits, Li is still not considered a trace element, and the main sources of this mineral in human diet are cereals and vegetables; thus, vegetarian diets may provide more Li than diets based on animal proteins. A study conducted in 2011 revealed that plant-based nuggets presented lower K, Zn, Cu, and Fe content than chicken nuggets^7^. An earlier study in 2006 showed higher K, Ca, and P in plant-based mince than in ground beef^7^. In the recent study conducted in major supermarkets^3^ of Sydney (Australia), differences in Na content were not consistent among plant-based products (burger, sausages, and mince). Although Fe content in our study was twice greater (P = 0.003) in PBB (26.51 mg/kg of the raw product) than in MBB (13.05 mg/kg of the raw product), it would be interesting to identify the proportion of heme-Fe in both burgers categories. In healthy conditions, the heme-Fe is much more easily absorbed from the diet than nonheme-Fe^17^. Therefore, it will be more relevant to identify the bioaccessibility and bioavailability of the minerals rather than the content itself, especially for potential toxic minerals (e.g. Cd) and for trace elements (e.g. Fe).

### Detailed protein profile

Although no differences were found in total protein content between burgers categories (PBB, Median 18.01% of the raw product; MBB, Median 17.96% of the raw product; S-Table 1), detailed protein profile showed that 5 out of the 18 amino acids identified in both samples differed significantly (S-Table 4). In contrast with our results, a lower protein content was reported in commercial plant-based products (burgers, sausages, and mince) compared with their meat homonymous^3^. Hydroxyproline is a major component of the protein collagen and was only identified in MBB category samples (S-Table 1). Thus, it can be used to identify animal-derived collagen and gelatin in food products^18^ and opens the possibility of using infrared spectroscopy models for rapid authentication of product origin. This technique has already demonstrated their potential application for the determination of other beef quality traits^19,20^. Alanine, glycine, and methionine were less abundant in PBB than in MBB, especially when referring to methionine, which was 21-fold lower. Methionine is one of the nine essential amino acids for human beings. On the contrary, cysteine and glutamic acid were more abundant in PBB than in MBB. In line with our results, ground meat analogue provided a relatively high protein profile, with the exception of methionine, compared to ground beef^7^. In fact, plant-based protein isolates, specifically from soy and pea –main ingredients of our PBB–, are particularly low in methionine content compared to animal-based proteins^21^. However, the use of plant-based protein blends may help reducing these differences^21^. As discussed for minerals content, the bioavailability of amino acids from PBB should be considered, since it is greater when coming from animal-based than from plant-based foods^22^. Nevertheless, the use of purified plant protein sources such as soy protein isolate or pea protein concentrate –which are in fact the ones used to manufacture the PBB included in our study– have a digestibility based on PDCAAS similar to that of animal-based protein sources^22^.

### Fatty acid profile

Although no differences were found in the total fat content between burger categories (PBB, Median, 11.10% of the raw product; MBB, Median, 12.51% of the raw product), PBB presented much lower (P = 0.003) cholesterol content (Median, 3.98 mg/100 g of the raw product) than MBB (Median, 50.60 mg/100 g of the raw product; S-Table 1). Those results partially agreed with the study conducted in plant-based and chicken nuggets that revealed lower cholesterol and total fat content in the former than in the latter^7^. On the other hand, lower content of total and saturated fat was detected in commercial plant-based products (burgers, sausages, and mince) compared to their meat homonymous^3^, whereas we obtained a similar saturated fat proportion in both burger categories (Table 2).

**Table 2:** Fatty acid (FA, median and 95% CI_50%_^1^) composition (% of total FAs) in the raw product.

Group^2^	Meat-based burger		Plant-based burger		*p*
	Median	95% CI_50%_	Median	95% CI_50%_	
SFAs	48.8	45.63–53.38	52.18	40.49–61.93	0.631
MUFAs	45.66	38.20–50.64	32.29	16.11–41.34	0.026
PUFAs	4.92	3.90–10.53	20.12	15.42–22.98	0.003
n-3	0.64	0.40–0.89	3.56	0.26–4.04	0.514
n-6	3.91	3.19–8.44	15.76	11.72–22.32	0.003
n-6/n-3 ratio	7.26	5.26–9.47	3.51	3.22–84.96	0.514
CLA	0.55	0.45–0.79	0.044	0.035–0.054	0.003
*cis*-FAs	2.82	2.35–2.46	0.93	0.32–1.85	0.004
*trans*-FAs	0.125	0.057–0.179	0.079	0.004–0.099	0.037
SCFAs	0.18	0.14–0.40	7.24	5.32–8.91	0.003
MCFAs	35.92	35.12–37.59	41.90	32.19–49.70	0.186
LCFAs	64.47	63.45–65.06	50.90	41.40–62.45	0.012

^1^95% CI_50%_: median 95% confidence interval.

^2^SFAs: saturated FAs; MUFAs: monounsaturated FAs; PUFAs: polyunsaturated FAs; CLA: conjugated linoleic acid; n-3: Omega-3 FAs; n-6: Omega-6 FAs; SCFAs: short-chain FAs; MCFAs: medium-chain FAs; LCFAs: long-chain FAs; *cis*-FAs: *cis* stereoisomers of FAs; *trans*-FAs: *trans* stereoisomers of FAs excluding CLA.

The greater content of polyunsaturated fatty acids in PBB than in MBB (Table 2), in particular of n-6 fatty acids, agreed with the results observed in plant-based mince and ground beef^7^. The n-6 fatty acids are associated with inflammation, constriction of blood vessels, and platelet aggregation, whereas n-3 fatty acids have opposite effects^23^. The greater n-6 content in PBB than in MBB (Table 2), particularly linoleic acid (C18:2n6; Table 3), was expected because crop seeds and vegetable oils such as sunflower oils are rich in linoleic acid and have a low proportion of n-3, specifically α-linolenic acid (18:3n3)^23^. Despite the greater variability in PBB than in MBB samples, α-linolenic acid proportion was statistically similar (P = 0.42) in both burger categories (PBB, Median, 3.48% of total fatty acids; 95% CI_50%_, 0.26–3.95% of total fatty acids; MBB, Median, 0.53% of total fatty acids; 95% CI_50%_, 0.30–0.81% total fatty acids), as well as the median of the n-6/n-3 ratio (Table 2). However, the n-3 and n-6/n-3 ratio variability was strongly influenced by one brand included in the PBB category, making it difficult to detect significant differences between both categories. The lower proportion of conjugated linoleic acid in PBB than in MBB (Table 2) was expected because meat and milk from ruminants, especially under grass-fed, are the main sources of conjugated linoleic acid in our diet^24^. Thus, conjugated linoleic acid has the potential to be used as an authentication tool to track down presence of cow, sheep, and goat meat in plant-based foods.

**Table 3:** Most abundant individual fatty acids (median and 95% CI_50%_^1^) expressed as percentage on total fatty acids in the raw meat- and plant-based burgers.

Fatty acid	Meat-based burger		Plant-based burger		*p*
	Median	95% CI_50%_	Median	95% CI_50%_	
C12:0	0.16	0.09–0.37	23.83	17.65–29.04	0.010
C14:0	3.13	3.07–3.36	9.60	7.23–11.36	0.003
C16:0	26.33	25.83–26.51	7.72	6.97–9.01	0.003
C18:0	15.41	12.92–19.29	2.37	2.23–3.06	0.003
C18:1n9	36.13	30.62–39.97	29.88	15.40–38.35	0.119
C18:2n6	2.65	2.04–6.46	15.66	11.64–22.19	0.003

^1^95% CI_50%_: median 95% confidence interval.

Despite the low proportion of *trans*-FAs in both burgers’ categories, MBB showed a greater content than PBB (Table 2). Dietary *trans*-FAs come mainly from industrially hydrogenated fats (up to 60% of total FAs content), mainly processed foods and oils, and a small amount (2% to 5%) from beef and dairy products due to the bacterial action in the rumen^25^. Hydrogenation process is used to provide firmness and plasticity to shortenings from vegetal oils –which are mainly composed of unsaturated fats–, enabling the production of solid and semisolid fats^25^. However, the PBB analyzed in the present study were elaborated with coconut oil (third major ingredient), that is a particular vegetal oil rich in saturated fats, which could explain the lower proportion of *trans*-FAs detected.

Both categories showed an expected greater content in long-chain fatty acids than in medium- and short-chain fatty acids^26^, particularly evident in MBB whose proportion of short-chain fatty acids was negligible (Table 2). In PBB, caprylic (C8:0) and capric (C10:0) acids were the most abundant short-chain fatty acids (Median 3.86% and 3.09%, respectively). These two fatty acids are found in high quantities in coconut oil^27^, which was the third major ingredient in all analyzed PBB. That ingredient could also explain the greater levels of lauric (C12:0) and myristic (C14:0) acids in PBB than in MBB (Table 3). On the other hand, the greater content of palmitic (C16:0) and stearic (C18:0) acids in MBB than in PBB could be linked to the great content of both fatty acids in the adipose and muscle tissue of animals^28^. Probably, the lack of significance for medium-chain fatty acids between categories is due to the fact that lauric, myristic, and palmitic acids were included in this same group. Although palmitic and stearic acids presented a lower proportion in PBB than in MBB, these are quite abundant in coconut oil and sunflower oil. The latter is listed as an ingredient in two of the PBB brands included in the present study.

The atherogenic and thrombogenic indices estimate the relation of diets to coronary heart disease^27^. In the present study, we obtained conflicting results. Whereas the atherogenic index was greater (P = 0.012) in PBB (Median, 1.47; 95% CI_50%_, 0.90–2.19) than in MBB (Median, 0.77; 95% CI_50%_, 0.70–0.84), the thrombogenic index was lower (P = 0.003) in PBB (Median, 0.60; 95% CI_50%_, 0.41–1.19) than in MBB (Median, 1.66; 95% CI_50%_, 1.48–1.19). However, these results can be explained by the use of coconut oil in the PBB manufacturing, which is a vegetal oil rich in saturated fatty acids, as previously discussed. In addition, the use of coconut and sunflower oils in the PBB manufacturing could also explain the lack of significant differences (P = 0.186) on the nutritional value between categories because (PBB: Median, 0.92; 95% CI_50%_, 0.59–1.32; MBB: Median, 0.71; 95% CI_50%_, 0.63–0.77), as already stated, these oils are rich in lauric, myristic, and palmitic acids, which are the fatty acids considered in the numerator of the formula. On the other hand, the hypocholesterolemic/Hypercholesterolemic ratio was greater (P = 0.003) in PBB (Median, 2.69; 95% CI_50%_, 1.85–4.16) than in MBB (Median, 1.48; 95% CI_50%_, 1.42–1.65) which indicates that PBB presented a greater content of fatty acids considered as hypocholesterolemic while the amount of hypercholesterolemic fatty acids are lower.

All the differences in the detailed chemical composition of PBB and MBB impact the burgers’ gross energy when calculated as dry matter (**DM**), being lower in PBB (Median, 24.86 MJ/kg DM) than in MBB (Median, 28.42 MJ/kg DM; P = 0.031) (S-Table 1). In fact, we observed lower long-chain fatty acids content in favor of shorter fatty acids, which have a slightly lower energy content than longer fatty acids^26^. Our results were in agreement with those obtained when comparing plant-based nuggets^7^ and plant-based products (burgers, sausages, and mince)^3^ with their meat homonymous.

## Conclusions

The results of the present study demonstrated the feasibility of introducing plant-based meat substitutes in our diet (i.e., usually richer in meat and processed products and ready-to-eat meals), given their interesting nutritional composition in protein profile, mineral, and fiber. However, both bioaccessibility and bioavailability of nutrients should be carefully considered to correctly estimate the nutritional value of plant-based foods and to properly compare them with their meat homonymous. The amino acids methionine and hydroxyproline might be used to authenticate ‘simulated beef’ products because methionine is more abundant in meat products and hydroxyproline is considered a signature acid in gelatin and collagen, and may open the possibility of applying infrared spectroscopy techniques with this scope. Conjugated linoleic acid might be also used to detect the presence of ruminant meat and fat in plant-based burgers. The use of coconut oil as an ingredient in plant-based burgers is responsible for the comparable saturated fat content to meat burgers. Flexitarian diets might be a more adequate approach to keep a balanced diet and to ensure the adequate intake of essential amino acids such as methionine and some specific fatty acids compared to a vegan or a more conventional occidental diet (processed products and ready-to-eat meals).

## Methods

### Sample collection

Samples from two burgers’ categories (PBB and MBB) were purchased to conduct the present study and, within each category, several brands were included. Three PBB products (9 per brand; n = 27) available to be purchased as raw product by consumers in the European Union market were included in the present study: Beyond burger (Beyond Meat, USA; Grocery store Metro di Padova, Padova, Italy), Incredible burger (Garden Gourmet, Nestlé, Switzerland; Grocery store Esselunga, Padova, Italy), and Planty of burger (Planty of meat, Frostmeat GmbH, Germany; Grocery store Metro di Padova, Padova, Italy). In addition, four MBB products (6 per brand; n = 24) were purchased in the grocery store Centro Carni Company Spa (Tombolo, Italy). All products were purchased in January 2020 in sealed commercial packages and brought to the food laboratory of the Department of Agronomy, Food, Natural resources, Animals and Environment of the University of Padova (Legnaro, Italy). Each burger was individually vacuum-packed in plastic bags and immediately frozen (–18 °C) until the analyses were performed. Prior to the analyses, all samples were thawed during 24 h at 4 °C and the plastic bag removed. Initial thawed weight (median) was 115.09 g (from 109.12 to 127.36; n = 27) for PBB and 152.58 g (147.86 g to 155.41; n = 24) for MBB.

Ingredients listed in the label of each product were:

- *Beyond burger*: pea protein isolate, expeller-pressed rapeseed oil, refined coconut oil, water, yeast extract, maltodextrin, natural flavors, gum arabic, sunflower oil, salt, succinic acid, acetic acid, non-GMO modified food starch, cellulose from bamboo, methylcellulose, potato starch, beet juice extract, ascorbic acid, apple extract, citrus fruit extract, vegetable glycerin.
- *Incredible burger*: water, soy protein, vegetable oils (rapeseed, coconut), natural flavors, wheat gluten, stabilizer (methylcellulose), vinegar, vegetable and fruit concentrates (beetroot, carrot, red capsicum, blackcurrant), salt, malt extract (barley).
- *Planty of burger*: water, protein concentrate (sunflower and pea), refined coconut oil, sunflower oil, onions, natural flavors, stabilizer (methylcellulose), lemon juice, vinegar, salt, food coloring, beetroot powder, bamboo fiber, smoked salt, sugar, acerola powder, maltodextrin.
- All four brands included in the meat burgers category were manufactured with the same ingredients: beef (85%), water, potato flakes, salt, vegetal fiber from citrus fruits, pea and carrot, natural flavors, antioxidant (ascorbic acid), spices.

### Color analysis and pH on the raw product

The L*, a*, b* components of each raw burger were assessed at room temperature (18.6 °C to 19.5 °C) with a Minolta CM-600d colorimeter (Minolta, Osaka, Japan) using the specular component included mode with 10° standard observer, D65 illuminant and an aperture of 8 mm, according to CIE-lab^30^. The pH was measured on the raw burgers with a Crison Basic 20 pH meter (Crison SpA, Carpi, Modena, Italy). The final value per sample was the average of 5 reads; two burgers per MBB brand (n = 8) and three burgers per PBB brand (n = 9) were analyzed.

### General composition of the raw product

Moisture was determined at 103 °C for 24 h (# 950.46), total protein as N × 6.25 using the Kjeldhal method (# 981.10), total lipids by the Soxhlet method with petroleum ether as solvent (# 991.36), and total ash was measured gravimetrically by igniting samples in a muffle furnace at 550 °C for 4 h (# 920.153) according to AOAC^31^. Total carbohydrates were calculated as: %carbohydrates = 100—(%moisture + %protein + %lipid + %ash). Collagen^32^ was calculated as: %collagen = (Hydroxyproline × 8)/10^3^.

Total dietary fiber was determined with an enzymatic–gravimetric method (# 991.43), and starch and sugars (# 996.11 and # 979.10, respectively) were determined with enzymatic digestion with amyloglucosidase followed by HPLC (Agilent 1260 Infinity) according to AOAC^31^. Results were expressed in ‘% of the raw product’. Cholesterol content was obtained with a capillary column GC system (model GC-15A, Shimadzu Corp., Kyoto, Japan) with previous saponification (# 976.26 and # 994.10) according to AOAC^31^. Results were expressed in mg/100 g of the raw product. Gross energy was determined with an isoperibolic-adiabatic calorimeter bomb (IKA C6000, Staufen, Germany) following the methodology ISO 9831:1998. Gross energy was expressed as MJ/kg in DM and MJ/kg of the raw product. All reads were performed in triplicate; two burgers per MBB brand (n = 8) and three or four burgers per PBB brand (n = 10) were analyzed.

### Cooking loss and Shear force

Cooking loss (**CL**) was measured as weight difference after cooking and expressed as percentage. It was calculated for both cooking methods: water bath and cooking plate. To determine CL using the water bath^33^, a complete burger was weighed, held in a plastic bag, and immersed for 60 min in a water bath at 75 °C to guarantee an internal temperature of 70 °C through a thermometer probe. Then, bags were cooled in water for 10 min and samples were extracted from the bags, blotted dry with paper towels, and weighed. For the determination of CL using the cooking plate, a complete burger was weighed and cooked using a flat electric hot plate made of Teflon at 200 °C for 10 min. Then, it was blotted dry with paper towels and weighed. For each method, diameter and height were also measured before and after cooking each sample to calculate the area and volume loss expressed as percentage. Determinations were performed in duplicate (i.e., two burgers) per MBB brand (n = 8) and in duplicate or triplicate (i.e., two or three burgers) per PBB brand (n = 9 for water bath method, and n = 8 for cooking plate method).

Shear force was measured with a Warner–Bratzler texture analyzer (LS5, Ametek Lloyd Instruments, Fareham, UK) equipped with Allo-Kramer shear10 blades. A 30-cm^3^ prim (7.5 × 4.0 × 1.0 cm) per cooked burger was obtained from each sample and cut with a force of 5000 N and a speed of 250 mm/min. Shear force was then calculated with the NEXYGEN Plus 3 software (Bognor Regis, UK) and expressed as N. Determinations were performed in duplicate (i.e., two burgers) per MBB brand (n = 8) and in duplicate or triplicate (i.e., two or three burgers) per PBB brand (n = 9 for water bath method and n = 8 for cooking plate method).

### Mineral profile of the raw product

Minerals were quantified after mineralization of the sample (0.5 g) with 7 ml of 67% nitric acid (**HNO**_**3**_) and 2 ml of 30% hydrogen peroxide in closed vessels by a microwave system (200 °C for 15–18 min, cooled to 35 °C, and made up to volume with distilled water; Ethos 1600 Milestone S.r.l. Sorisole, Bergamo, Italy) using inductively coupled plasma optical emission spectrometry (**ICP-OES**) Arcos EOP (SPECTRO Analytical Instruments GmbH, Kleve, Germany) according to AOAC^31^ method # 2013.06. Wavelengths used to determine each mineral were: Ag at 328.068 nm, Al at 167.078 nm, As at 189.042 nm, B at 208.959 nm, Ba at 455.404 nm, Be at 313.042 nm, Ca at 315.887 nm, Cd at 214.438 nm, Co at 228.616 nm, Cr at 205.618 nm, Cu at 324.754 nm, Fe at 259.941 nm, Hg at 184.950 nm, K at 766.941 nm, Li at 670.780 nm, Mg at 285.213 nm, Mn at 257.611 nm, Mo at 202.095 nm, Na at 589.592 nm, Ni at 231.604 nm, P at 177.495 nm, Pb at 220.353 nm, S at 182.034 nm, Sb at 206.833 nm, Se at 196.090 nm, Si at 251.612 nm, Sn at 189.991 nm, Sr at 407.771 nm, Te at 214.281 nm, Ti at 334.941 nm, Tl at 190.864 nm, V at 311.071 nm, and Zn at 213.856 nm. Instrument operating parameters were optimized for acid solution and calibration standards were matched with 5% HNO_3_ (v/v) solution using 65% HNO_3_ Suprapur® (100,441, Merck, Darmstadt, Germany). Operating conditions of ICP-OES were 2 mL/min of sample aspiration rate, plasma power 1350 W, coolant flow 12 L/min, auxiliary flow 0.80 L/min, nebulizer flow 0.90 L/min, and integration time of 28 s. The final value per sample was the average of 3 reads; two burgers per MBB brand (n = 6) and three or four burgers per PBB brand (n = 10) were analyzed. The calibration solutions for each mineral were prepared from single element solutions (Inorganic Ventures, Christiansburg, VA, USA) in a concentration range between 0 and 100 mg/L. Minerals were expressed in mg/kg of the raw product.

### Amino acid profile of the raw product

Amino acids were analyzed after acid hydrolysis and pre-column derivatization with6-aminoquinolyl-N-hydroxysuccinimidyl carbamate (AQC), separated by RP-HPLC and analyzed by UV detection^34^. Briefly, for Ala, Arg, Asp, Glu, Gly, Hydroxyproline, Ile, His, Leu, Lys, Met, Phe, Pro, Ser, Tyr, Thr, and Val determination, protein of the samples was hydrolyzed with hydrochloride acid (6 M) at 105 °C for 24 h. The Cys was determined as sum of cysteine and cystine, after reaction with dithiodipropionic acid, producing a mixed disulphite, which then underwent acid hydrolysis accordingly. After hydrolysis, the samples were neutralized with sodium hydroxide (8 M), adjusted to volume and filtered at 0.45 µm. Then, the derivatization step was conducted according to the manufacturer’s instructions (AccQTag Ultra Derivatization Kit; Waters Corporation, Milford, MA, USA). The Trp was determined following CD 2000/45/EC^35^ indications using a basic hydrolysis with barium hydroxide at 105 °C for 24 h and after neutralization and filtration analyzed directly by RP-HPLC. Separation and quantification of amino acids were performed using an Agilent 1260 Infinity HPLC (Agilent Technologies, Santa Clara, CA, USA) equipped with a reversed-phase column C18 (CORTECS C18 Column, 90 Å, 2.7 μm, 250 mm × 2.1 mm; Waters Corporation, Milford, MA, USA) kept at 45 °C, and with a diode array Detector (Agilent 1260 Series, DAD VL +). The final value per sample was the average of 3 reads; two burgers per MBB brand (n = 6) and three or four burgers per PBB brand (n = 10) were analyzed. Results were expressed in mg/100 g of the raw product.

### Fatty acids profile of the raw product

For the fatty acids profiling, total lipids were extracted by an accelerated solvent extraction method using a Dionex ASE 350 system (Thermo Scientific, Dreieich, Germany) with petroleum ether as solvent (#960.39)^31^. Total fat content was determined after solvent evaporation with a rotavapor at 45 °C and expressed as a percentage. Fatty acid methyl esters of total lipids were prepared with an internal method adapted from Christie^36^. Briefly, for 40 mg of extracted fat, 1 mL of sulphuric acid in methanol was added, and samples were placed in an oven at 65 °C overnight. At the end of the methylation, 2 mL of n-heptane and 1 mL of potassium carbonate were added. Fatty acid methyl esters solutions were centrifuged for 10 min at 4000 × g at 4 °C and the supernatant was collected in a 1.5-mL vial.

Separation and quantification of fatty acids methyl ester were performed using an Agilent 7820A GC System equipped with an automatic sampler G4567A (Agilent Technologies, Santa Clara, CA, USA) and a flame ionization detector. The capillary column (length 30 m, inner diameter 0.25 mm, film thickness 0.25 μm) comprised an Omegawax capillary GC column (24,136 Supelco; Sigma-Aldrich, Castle Hill, Australia). The carrier gas was hydrogen at flow rate of 1.4184 mL/min with an average speed of 39.5 cm/s. The injector and temperature detector were both set at 250 °C. The oven temperature was initially 50 °C for 2 min and then increased at 4 °C/min until reaching 220 °C, at which point this temperature was held for 18 min. The individual fatty acids were identified by comparing their retention times with those of a standard fatty acid (Supelco FAME mixC4–C24 #18,919-1AMP; Sigma-Aldrich). Peaks areas were calculated using GC/MSD ChemStation Software (Agilent Technologies) and expressed as percentage of total fatty acids. The final value per sample was the average of 3 reads; two burgers per MBB brand (n = 6) and three or four burgers per PBB brand (n = 10) were analyzed.

The following fatty acids (**FA**s) groups were obtained by summing up individual FAs: saturated FAs, which included C4:0, C6:0, C8:0, C9:0, C10:0, C11:0, C12:0, C13:0, C14:0 (and iso and anteiso form), C15:0 (and iso and anteiso form), C16:0 (and iso and anteiso form), C17:0 (and iso and anteiso form), C18:0 (and iso and anteiso form), C19:0, C20:0, C21:0, C22:0, C23:0, C24:0; monounsaturated FAs, which included C10:1, C12:1 (all C12:1), C14:1 (all C14:1), C15:1t, C16:1 (all c16:1), C17:1 (all C17:1), C18:1 (all C18:1), C20:1n9, C22:1n9 and C24:1n9; polyunsaturated FAs, which included C16:2, C18:2n6, conjugated linoleic acid (all CLA isomers), C18:3 (C18:3n6 and C18:3n3) C18:4, C20:2n6, C20:3 (C20:3n6 and C20:3n3), C20:4n6, C20:5n3, and C22:6n3; CLA, which included geometric isomers of C18:2; **n-3**, which included omega-3 FAs; and **n-6**, which included omega-6 FAs; Short-chain FAs which included from C4:0 to C10:1; Medium-chain FAs which included from C11:0 to C16:2; Long-chain FAs which included from C17:0 to C22:6n3; cis FAs, which included all cis-isomers (*cis* C18:1n7) except within CLA; trans FAs, which included all trans-isomers (*trans* C12:1, *trans* C14:1, *trans* C15:1, *trans* C16:1, *trans* C18:2n6) except within CLA.

Moreover, the atherogenic index (**AI**) and the thrombogenic index (**TI**) were calculated applying the formula proposed by Ulbricht and Southgate^29^:

$$\begin{aligned} {\text{AI}} & = \left[ {{\text{C}}12:0 + \left( {4 \times {\text{C}}14:0} \right) + {\text{C}}16:0} \right]/{\text{UFAs}} \\ {\text{TI}} & = \left( {{\text{C}}14:0 + {\text{C}}16:0 + {\text{C}}18:0} \right)/[(0.5 \times {\text{MUFAs}}) + \left( {0.5 \times {\text{n}}6} \right) + \left( {3 \times {\text{n}}3} \right) + \left( {{\text{n}}3/{\text{n}}6} \right)] \\ \end{aligned}$$

the nutritional value (**NV**) was calculated applying the formula proposed by Estévez, Morcuende, Ramírez, Ventanas and Cava^37^:

$${\text{NV}} = \left( {{\text{C}}12:0 + {\text{C}}14:0 + {\text{C}}16:0} \right)/\left( {{\text{C}}18:1{\text{n}}9 + {\text{C}}18:1{\text{n}}7 + {\text{C}}18:1{\text{n}}5 + {\text{C}}18:2{\text{n}}6} \right)$$

the hypocholesterolemic/Hypercholesterolemic ratio (**h/H**) ratio was calculated applying the formula proposed by Fernández et al*.*^38^:

$$ \begin{aligned}{\text{h}}/{\text{H}} &= \left( {{\text{C}}18:1{\text{n}}9 + {\text{C}}18:1{\text{n}}7 + {\text{C}}18:1{\text{n}}5 + {\text{C}}18:2{\text{n}}6 + {\text{C}}18:3{\text{n}}3 + {\text{C}}18:3{\text{n}}6 + {\text{C}}18:4{\text{n}}3 + {\text{C}}20:1{\text{n}}9 + {\text{ C}}20:2{\text{n}}6}\right.\\&\quad  \left.{ + {\text{C}}20:3{\text{n}}6 + {\text{C}}20:3{\text{n}}3 + {\text{C}}20:4{\text{n}}6 + {\text{C}}20:5{\text{n}}3 + {\text{C}}22:1{\text{n}}9 + {\text{C}}22:6{\text{n}}3 + {\text{C}}24:1{\text{n}}9} \right)/\left( {{\text{C}}14:0 + {\text{C}}16:0} \right).\end{aligned} $$

### Statistical analysis

Data showed a non-normal distribution which was not normalized using Box-Cox transformations^39,40^. As a consequence, the category of burger (MBB vs PBB) effect was analyzed using a non-parametric Mann–Whitney U test (PROC NPAR1WAY) in the SAS software version 9.4 (SAS Institute Inc., Cary, NC, USA). Data are reported as median with the median 95% confidence interval (**95%** CI_**50%**_). Significance was established at P < 0.05.

## Supplementary Information

Supplementary Information 1.
