# Supplementary material for: Detailed characterization of plant-based burgers
Source: Sci Rep. 2021 Jan 21;11:2049. doi: 10.1038/s41598-021-81684-9 (PMC7820238; doi:10.1038/s41598-021-81684-9)
Supplement: Supplementary file 1 — Supplementary Information 1. [file 41598_2021_81684_MOESM1_ESM.pdf]

Detailed characterization of plant-based burgers

Massimo De Marchi*, Angela Costa, Marta Pozza, Arianna Goi, Carmen L. Manuelian*

Department of Agronomy, Food, Natural resources, Animals and Environment, University of Padova, Viale dell'Università 16, 35020 Legnaro (PD), Italy

*corresponding authors: massimo.demarchi@unipd.it; carmenloreto.manuelianfuste@unipd.it

Supplementary Information Tables Legends

S-Table 1. Gross composition (of the raw product) and gross energy (of the raw product and in DM). Results are reported as median and 95% $CI_{50\%}$[1].

S-Table 2. Cooking loss (%), volume loss (%), and shear force (N) of cooked burgers in water bath or in plate. Results are reported as median and 95% $CI_{50\%}$[1].

S-Table 3. Mineral composition (mg/kg of the raw product). Results are reported as median and 95% $CI_{50\%}$[1].

S-Table 4. Amino acids composition (mg/100 g of the raw product). Results are reported as median and 95% $CI_{50\%}$[1].

S-Table 1. Gross composition (of the raw product) and gross energy (of the raw product and in DM). Results are reported as median and 95% $CI_{50\%}$[1].

Item	Meat-based burger		Plant-based burger		p
	Median	95% $CI_{50\%}$	Median	95% $CI_{50\%}$	
Moisture (%)	65.91	61.51-69.60	60.91	52.81-64.01	0.037
Ashes (%)	1.79	1.77-1.82	2.52	1.87-3.47	0.003
Protein (%)	17.96	15.89-18.75	18.01	13.30-18.43	0.514
- Collagen[2] (%)	2.49	1.37-2.83	-	-	-
Fat (%)	12.51	7.98-20.33	11.10	8.76-19.08	0.631
- Cholesterol (mg/100 g of the raw product)	50.60	48.81-54.26	3.98	3.88-4.55	0.003
Carbohydrates (%)	2.09	2.00-2.76	8.37	7.62-10.03	0.003
- Starch (%)	0.93	0.86-1.14	0.31	0.10-1.21	0.161
- Fructose (%)	0.022	0.019-0.055	0.013	0.010-0.056	0.571
- Total dietary fiber (%)	0.74	0.48-0.98	4.27	2.90-5.02	0.003
Gross energy (MJ/kg DM)	28.42	26.11-30.26	24.86	23.98-28.61	0.031
Gross energy (MJ/kg of the raw product)	9.69	7.94-12.19	9.41	8.92-13.50	1.00

[1]95% $CI_{50\%}$: median 95% confidence interval

[2]Collagen percentage was calculated as (Hydroxyproline × 8)/10^3

S-Table 2. Cooking loss (%), volume loss (%), and shear force (N) of cooked burgers in water bath or in plate. Results are reported as median and 95% $CI_{50\%}$[1].

Trait	Meat-based burger		Plant-based burger		p
	Median	95% $CI_{50\%}$	Median	95% $CI_{50\%}$	
Cooking loss[2] (%)	25.67	24.15-32.47	16.01	8.55-20.41	0.004
Volume loss[2] (%)	26.01	17.89-34.41	20.23	13.50-25.02	0.094
Shear force[2] (N)	12.85	10.70-16.89	6.34	5.92-6.67	0.004
Cooking loss[3] (%)	23.06	21.15-29.16	21.04	18.79-26.11	0.071
Volume loss[3] (%)	27.96	25.32-48.13	21.84	14.24-35.12	0.246
Shear force[3] (N)	13.70	10.57-18.27	10.10	8.78-15.01	0.086

[1]95% $CI_{50\%}$: median 95% confidence interval

[2]Using the water bath method

[3]Using the cooking plate methods

S-Table 3. Mineral composition (mg/kg of the raw product). Results are reported as median and 95% $CI_{50\%}$[1].

Mineral	Meat-based burger		Plant-based burger		p
	Median	95% $CI_{50\%}$	Median	95% $CI_{50\%}$	
Major minerals					
Na	4267.44	4214.72-4445.18	4284.66	3653.16-7152.97	1.000
K	2717.57	2524.12-2959.74	3457.13	3279.45-5448.62	0.003
P	1270.13	1191.41-1415.95	2098.92	1558.16-3647.59	0.003
S	1325.11	1297.02-1537.88	1442.76	1113.35-1692.15	0.460
Mg	159.42	151.84-174.21	614.94	181.17-1404.44	0.003
Ca	85.62	79.72-141.48	715.39	176.82-853.47	0.003
Trace minerals					
Zn	30.69	27.61-36.33	21.38	8.35-25.11	0.003
Si	20.89	19.26-23.61	20.43	16.21-98.81	0.965
Fe	13.05	11.62-14.14	26.51	23.93-33.09	0.003
Cu	1.52	0.80-4.28	3.52	2.62-9.12	0.009
Sr	0.50	0.46-0.53	2.76	0.61-3.03	0.007
Mn	0.18	0.17-0.27	10.52	2.96-11.30	0.003
Li[2]	0.10	-	0.28	0.26-0.38	-
Cr	0.08	0.07-0.11	0.18	0.15-0.31	0.003
Ba	0.07	0.06-0.26	0.53	0.14-2.36	0.005
Ni	0.06	0.06-0.10	0.22	0.19-0.33	0.003
Ti	0.05	0.04-0.07	0.17	0.11-0.81	0.003

B[3]	-	-	0.79	0.15-4.14	-
Mo	-	-	0.22	0.10-0.88	-
Heavy metals					
Cd[4]	-	-	0.14	0.14-0.15	-

[1]95% $CI_{50\%}$: median 95% confidence interval

[2]Li was only detected in 1 sample of meat-based burgers, and in 4 samples of the same plant-based burgers brand

[3]B was only detected in 9 samples of plant-based burgers

[4]Cd was only detected in 4 samples of plant-based burgers of the same brand

S-Table 4. Amino acids composition (mg/100 g of the raw product). Results are reported as median and 95% $CI_{50\%}$[1].

Amino acid	Meat-based burger		Plant-based burger		P-value
	Median	95% $CI_{50\%}$	Median	95% $CI_{50\%}$	
Alanine	1096.24	815.93-1214.69	686.97	605.63-818.55	0.007
Arginine	1085.62	912.53-1489.29	1060.73	930.21-1478.36	0.965
Aspartic acid	1581.08	1232.11-1885.85	1925.04	1549.21-2502.18	0.062
Cysteine	154.13	141.88-187.69	251.91	174.84-308.23	0.012
Glycine	1305.27	949.08-1910.00	689.43	640.20-761.76	0.003
Glutamic acid	3027.17	2275.92-3515.55	4351.61	4017.72-5805.17	0.003
Hydroxyproline	310.93	171.16-354.14	-	-	-
Isoleucine[2]	554.97	463.71-749.81	507.7	472.27-618.83	0.631
Histidine[2]	582	498.67-773.87	591.48	417.03-869.42	0.965
Leucine[2]	1164.39	962.76-1496.66	1214.58	872.24-1500.31	0.965
Lysine[2]	1391.19	1046.08-1836.15	927.82	707.09-1604.09	0.138
Methionine[2]	300.11	243.62-392.31	13.76	9.64-51.06	0.003
Phenylalanine[2]	661.94	613.06-891.79	899.28	630.23-1066.58	0.101
Proline	855.61	696.31-1047.03	774.66	534.19-1159.60	0.631
Serine	672.63	535.50-884.56	902.61	593.11-1038.95	0.215
Tyrosine	481.56	436.45-642.64	591.16	365.79-675.24	0.760
Threonine[2]	686.22	579.13-927.21	572.28	468.41-647.78	0.062
Tryptophan[2]	102.51	93.19-133.41	108.28	87.09-128.34	0.965
Valine[2]	612.10	575.45-825.46	559.02	516.81-671.49	0.215

[1]95% $CI_{50\%}$: median 95% confidence interval

[2]Essential amino acids
